# Identification of Biomarkers for Esophageal Squamous Cell Carcinoma Using Feature Selection and Decision Tree Methods

**DOI:** 10.1155/2013/782031

**Published:** 2013-12-12

**Authors:** Chun-Wei Tung, Ming-Tsang Wu, Yu-Kuei Chen, Chun-Chieh Wu, Wei-Chung Chen, Hsien-Pin Li, Shah-Hwa Chou, Deng-Chyang Wu, I-Chen Wu

**Affiliations:** ^1^School of Pharmacy, Kaohsiung Medical University, Kaohsiung 80708, Taiwan; ^2^Ph.D. Program in Toxicology, Kaohsiung Medical University, Kaohsiung 80708, Taiwan; ^3^Department of Family Medicine, Kaohsiung Medical University Hospital, Kaohsiung 80708, Taiwan; ^4^Department of Public Health, Kaohsiung Medical University, Kaohsiung 80708, Taiwan; ^5^Center of Environmental and Occupational Medicine, Kaohsiung Municipal Hsiao-Kang Hospital, Kaohsiung 812, Taiwan; ^6^Department of Food Science and Nutrition, Meiho University, Pingtung 91202, Taiwan; ^7^Department of Pathology, Kaohsiung Medical University Hospital, Kaohsiung 80708, Taiwan; ^8^Division of Chest Surgery, Kaohsiung Municipal Hsiao-Kang Hospital, Kaohsiung 812, Taiwan; ^9^Division of Chest Surgery, Department of Surgery, Kaohsiung Medical University Hospital, Kaohsiung 80708, Taiwan; ^10^Division of Gastroenterology, Department of Internal Medicine, Kaohsiung Medical University Hospital, Kaohsiung 80708, Taiwan; ^11^Department of Medicine, Faculty of Medicine, College of Medicine, Kaohsiung Medical University, Kaohsiung 80708, Taiwan

## Abstract

Esophageal squamous cell cancer (ESCC) is one of the most common fatal human cancers. The identification of biomarkers for early detection could be a promising strategy to decrease mortality. Previous studies utilized microarray techniques to identify more than one hundred genes; however, it is desirable to identify a small set of biomarkers for clinical use. This study proposes a sequential forward feature selection algorithm to design decision tree models for discriminating ESCC from normal tissues. Two potential biomarkers of RUVBL1 and CNIH were identified and validated based on two public available microarray datasets. To test the discrimination ability of the two biomarkers, 17 pairs of expression profiles of ESCC and normal tissues from Taiwanese male patients were measured by using microarray techniques. The classification accuracies of the two biomarkers in all three datasets were higher than 90%. Interpretable decision tree models were constructed to analyze expression patterns of the two biomarkers. RUVBL1 was consistently overexpressed in all three datasets, although we found inconsistent CNIH expression possibly affected by the diverse major risk factors for ESCC across different areas.

## 1. Introduction

Esophageal cancer is the sixth most common fatal human cancer in the world [1]. The histological type of esophageal squamous cell carcinoma (ESCC) is also one of the most common cancers in the Chinese population [2]. ESCC occurs more frequently in males than females [1]. As an aggressive tumor, the prognosis of ESCC is very poor because it is typically diagnosed after the presence of symptoms. The 5-year survival rate of ESCC patients is 19%: the fourth worst among all cancers in the USA [3].

Early detection of ESCC could be a promising strategy to decrease mortality. Microarray techniques are extensively utilized to measure expression levels of a large number of genes simultaneously and provide better understanding of the molecular mechanism of ESCC carcinogenesis. The microarray expression data could be analyzed to identify and give insights into clinical biomarkers of ESCC for detection. Several efforts have been made to study gene expression profiles and differential expressed genes for discovering biomarkers using microarray techniques [4–7]. Usually, more than one hundred genes are identified as either upregulated or downregulated genes. However, for clinical use, a small set of genes capable of distinguishing ESCC from normal tissues is much more useful. Thus the identification of a small number of biomarkers is desirable for ESCC detection.

The incorporation of classification and feature selection algorithms has been widely used to identify promising features for various classification problems such as ubiquitylation sites [8], immunogenic peptides [9], pupylation [10], and sumoylation sites [11]. The application of feature selection algorithm for cancer biomarker identification could give better insights into the mechanisms. For example, gene Signature Finder Algorithm has been recently proposed to identify biomarkers in colorectal cancer [12]. Feature selection algorithms can remove irrelevant genes and identify important genes to improve classification performance.

For the application of biomarkers for detecting ESCC, the simple decision tree methods capable of generating human interpretable rules were chosen instead of the black-box methods such as support vector machines (SVM). In this study, a sequential forward feature selection algorithm is proposed to identify genes best for decision tree classifications that is capable of selecting a small set of biomarkers with human interpretable rules. Two public available microarray datasets obtained from Gene Expression Omnibus (GEO) database [13] are utilized to identify and validate the biomarkers, respectively. Furthermore, 34 microarray experiments of 17 pairs of ESCC and normal tissues from Taiwanese male patients are performed to test the discrimination ability of the identified biomarkers. Results show that RUVBL1 (RuvB-like 1) and CNIH (Cornichon homolog) genes are useful for discriminating ESCC from normal tissues with a leave-one-out cross-validation accuracy of 91.18%.

## 2. Materials and Methods

### 2.1. Datasets

In order to identify and validate genomic biomarkers for ESCC, two microarray datasets of GSE23400 [6] and GSE20347 [7] were downloaded from Gene Expression Omnibus (GEO) database [13]. Both microarray experiments were performed on the Affymetrix Human U133A platforms consisting of expression profiles of ESCC and surrounding normal tissues of ESCC patients in China and were normalized using the Robust Multiarray Average (RMA) algorithm [14, 15] in Bioconductor available at http://www.bioconductor.org/. There are 18,400 transcripts and variants in Affymetrix Human U133 set, including approximately 14,500 well-characterized human genes in greater than 22,000 probe sets and 500,000 distinct oligonucleotide features. GSE23400 consisting of 53 ESCC and 53 normal tissues from patients was utilized to identify genomic biomarkers for ESCC. The GSE20347 consisting of 17 ESCC and 17 normal tissues was applied to validate the identified genomic biomarkers.

### 2.2. Study Subjects and RNA Isolation from Tumor Tissue and Adjacent Normal Tissue

We selected 17 incident male ESCC patients who regularly consumed tobacco and alcoholic beverage to validate the candidate biomarkers obtained from the above two datasets. All of them underwent total esophagectomy in Kaohsiung Medical University Hospital. One pair of resected tumor and adjacent normal tissue for each patient was immediately put into a portable container with dry ice and then transferred and maintained in a nitrogen tank until analysis. After review by a qualified pathologist (Dr. CC Wu), the tumor parts were found to have cancer cells in >80% of the tissues, whereas the normal parts were microscopically tumor-free. This study was in compliance with the Helsinki Declaration and approved by the internal review board of KMUH. All patients provided their written informed consent.

Total RNA from each pair was isolated by a single-step guanidinium isothiocyanate method using the Trizol Reagent total RNA Purification Kit (Invitrogen Inc., USA) according to the manufacturer's instructions. The yield and quality of RNA were assessed by spectrophotometry and the Agilent 2100 Bioanalyzer (Agilent Technologies, Palo Alto, CA). All paired samples had an A260/A280 between 1.8 and 2.2 and A260/A230 ratio above 1.0 and were eligible for the subsequent array experiment.

### 2.3. Reverse Transcription

After RNA isolation, cDNA was prepared from each sample by Reverse Transcription System (Cat: A3500, Promega Corporation, USA). For the appropriate efficiency of reverse transcription, 1 *μ*g total RNA was placed in a microcentrifuge tube and incubated at 70°C for 10 minutes in a thermocycler (Gene Amp PCR system 9700, Applied Biosystems). After denaturing secondary structure of RNA, the thermocycler was rapidly set at 4°C waiting for the next step. During the RNA denatured process, the reaction components were prepared according to the manufacturer's guideline. The final concentrations of reaction component were 5 mM MgCl_2_; 1X reverse transcription buffer (10 mM tris-HCl (pH 9.0 at 25°C); 50 mM KCl; 0.1% triton X-100); 1 mM each dNTP; 1 u/*μ*L recombinant RNasin ribonuclease inhibitor; 15 u/*μ*g AMV reverse transcriptase (high conc.); and 0.5 *μ*g oligo (dT)_15_. The reaction mixture was loaded into a microcentrifuge tube mixed with total RNA and incubated at 42°C for 15 minutes. Subsequently, the reaction mixture was heated to 95°C for 5 minutes for inactivating AMV reverse transcriptase and stored the first-strand cDNA at −20°C until analysis.

### 2.4. Human One-Array System

Human oligonucleotide DNA microarrays (Human Whole Genome OneArray) from Phalanx Biotech Group (Hsinchu, Taiwan) were used. The Human Whole Genome OneArray (HOAv4.3, Phalanx Biotech Group, Taiwan) contains 32,050 60-mer oligonucleotide probes, including 28,703 probes corresponding to the annotated genes in Unigene v175 and RefSeq database, 2,265 experimentally defined probes, and 1,082 control probes.

#### 2.4.1. Microarray Experiment

The detailed experimental method is described elsewhere [16]. Each cDNA sample from the paired tumor and normal parts of the 17 ECC patients was hybridized; thus, a total of 34 chips were used in this study. After nonspecific binding targets were washed, the hybridization arrays were conjugated with fluorescent detector of Streptaavidin-Cy3. Finally, arrays were dried by centrifugation and scanned by DNA Microarray Scanner (Agilent Technologies, Santa Clara, US). Images from the scanned arrays were quantified using GenePix Pro 4.0 (Molecular Devices, Sunnyvale, CA).

#### 2.4.2. Qualification and Normalization of Microarray Chips

Spots in each array with foreground median intensity of wavelength 532 nm greater than or equal to that of background median intensity plus 3-fold standard deviation of wavelength 532 nm were considered as the “Present” flag and included for the further analysis. In order to evaluate the quality of each array in the entire array experiment, three evaluation steps were performed: basic, reproducible, and diagram. In the basic step, three parameters, including percentage of “Present” spots among all spots, the average intensity of “Present” spots, and coefficient of variation of intensity for control spots in the entire arrays, were all considered. If any two parameters in one array were located outside the 1.5-folds interquartile range (25th–75th) of the same parameters for all arrays, that array was excluded. The remaining arrays were then evaluated in reproducible steps which the repeated arrays of the same sample would pass, when their Pearson's correlation coefficient was larger than 0.95 and “2-fold percentage” was less than 15%. The “2-fold percentage” was the percentage of probes among all probes in which the ratio of the same probe between two arrays exceeded 2-fold. In the final diagram step, the density plot of repeated arrays was used to examine the intensity profile of each array. An array would pass if the profile was similar to the rest of arrays in the same phenotype groups. When the arrays passed all three steps, the raw intensity of spots was log-2 transformed for subsequent analysis. To adjust the systematic variation of experiments and dye effects, global Lowess normalizations were performed within repeated arrays of the same sample and between the samples. Spot was included for further analysis when it was “Present” in at least one of the qualified arrays.

### 2.5. Decision Tree Algorithm

Decision tree algorithms are useful methods to generate interpretable rules based on gene expressions for ESCC classification that are widely used in various classification and regression problems such as immunogenic peptides [17], promoters [18], and nongenotoxic hepatocarcinogenicity [19]. In this study, a decision tree method J48 implemented in WEKA [20], also known as C4.5 [21], is applied to construct decision tree classifiers and derive interpretable rules. The construction of a decision tree is described as follows. First, information gain is utilized to rank features. Second, the top ranking features are iteratively appended as nodes to split data into subsets. The tree growing process stops when the data subset in each leaf node belongs to the same class. The fully grown tree is prone to overfit the training data. Therefore, a pruning process is applied to reduce the tree size by replacing a subtree with a leaf node to avoid overfitting problems. The pruning process is based on a default threshold value of 25% confidence. The samples in the leaf node are the covered samples of this rule. The class label of a leaf node is determined by a majority rule. The samples with a relative small size in the leaf node are regarded as misclassified samples. The final decision tree can directly generate if-then rules where one leaf node corresponds to one rule.

### 2.6. Sequential Forward Feature Selection

There are more than forty thousand probes in a microarray experiments. The selection of informative probes for discriminating between ESCC and normal tissues is a crucial step for biomarker identification. Although the decision tree algorithm J48 has a built-in function for feature selection, the incorporation of various feature selection algorithms could generate decision trees with higher classification accuracy [22, 23].

In this study, a sequential forward feature selection algorithm (SFFS) is proposed to identify useful biomarkers for discriminating between ESCC and normal tissues. The selection process is based on the accuracy of leave-one-out cross-validation (LOOCV) using the decision tree algorithm J48. Given a dataset of sample size *n*, *n* − 1 sample is utilized to train a decision tree classifier and the remaining one sample is utilized to validate the decision tree classifier for each run of LOOCV. The accuracy of LOOCV is calculated by averaging the *n* validation accuracies. Given an empty pool of selected probes *S*, the SFFS algorithm iteratively selects informative probes into *S* as shown as follows. First, the LOOCV accuracy is evaluated for each probe. Second, the best probes with highest LOOCV accuracy are appended into *S*. Third, for each remaining probe *p*, its LOOCV accuracy is evaluated by using *p* and probes in *S*. Fourth, the second and third steps are repeated until the termination criteria are satisfied. The resulting probe set *S* consists of the final biomarkers.

The SFFS algorithm utilizes the greedy selection strategy under the property monotonic assumption. In contrast to univariate feature selection methods, the SFFS algorithm considering the interaction effects of sequential selected probes on the accuracy is expected to perform better. The SFFS algorithm is only applied to training dataset to identify potential biomarkers.

### 2.7. Performance Measurement

To evaluate classifiers for their prediction performance, the leave-one-out cross-validation method is applied as it is widely used as an objective evaluation method for error rate estimation [8, 9, 24]. Three measurements were applied to evaluate classifiers including sensitivity, specificity, and accuracy defined as follows: sensitivity = TP/(TP + FN), specificity = TN/(TN + FP), and accuracy = (TP + TN)/(TP + FP + FN + TN), where TP, FP, FN, and TN are the numbers of true positives, false positives, false negatives, and true negatives, respectively. In this work, accuracy is used as major indicator for estimating the performance of classifiers.

## 3. Results 

### 3.1. Identification of Potential Biomarkers for ESCC

To identify potential biomarkers for ESCC, a microarray dataset GSE23400 is fetched from GEO database for the following analysis. GSE23400 consists of 53 pairs of ESCC and adjacent normal tissue from 53 patients in China. For each probe, *t*-test with multiple test correction of Benjamini and Hochberg is applied to calculate its corresponding *t*-statistic and adjusted *P* value using GEO2R [13]. The correction method of Benjamini and Hochberg providing a good balance between discovery of statistically significant genes and limitation of false positives is a commonly used adjustment for microarray data [25].

Because overexpressed genes are more useful for clinical diagnosis than downexpressed genes, only significantly differential expressed probes with adjusted *P* values smaller than 0.001 and overexpressed in tumor tissue were selected for subsequent analysis. A total of 3,910 probes were obtained by the above criteria.

To identify potential biomarkers for discriminating ESCC from adjacent normal tissues, a sequential forward feature selection (SFFS) algorithm is proposed to determine the best probe set giving the highest leave-one-out cross-validation (LOOCV) accuracy using a decision tree algorithm J48. By applying the proposed SFFS algorithm, two probes giving the highest LOOCV accuracy were selected as potential biomarkers whose gene names are RUVBL1 and CNIH.

**Figure 1:**
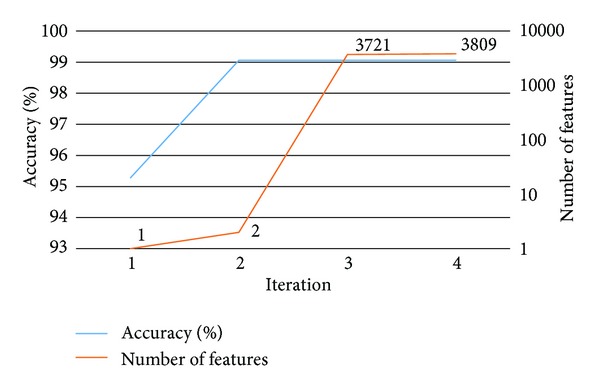
Selection results of the sequential forward feature selection algorithm.

The selection process of SFFS is shown in Figure 1. The first iteration of SFFS selects one gene RUVBL1 with an LOOCV accuracy of 95.28%. CNIH is selected in the second iteration of SFFS. The combined use of RUVBL1 and CNIH yields a higher accuracy of 99.06%. The third and fourth iterations select 3721 and 3905 genes without any further improvement in LOOCV accuracy. For further validation of the selected biomarkers, 10-fold cross-validation (10-CV) is applied to evaluate the classification performances. The 10-CV accuracies of RUVBL1 alone (95.28%) and combination of RUVBL1 and CNIH (97.17%) are equal to and slightly worse than the LOOCV accuracies, respectively, showing the usefulness of the biomarkers when less training samples are available.

**Figure 2:**
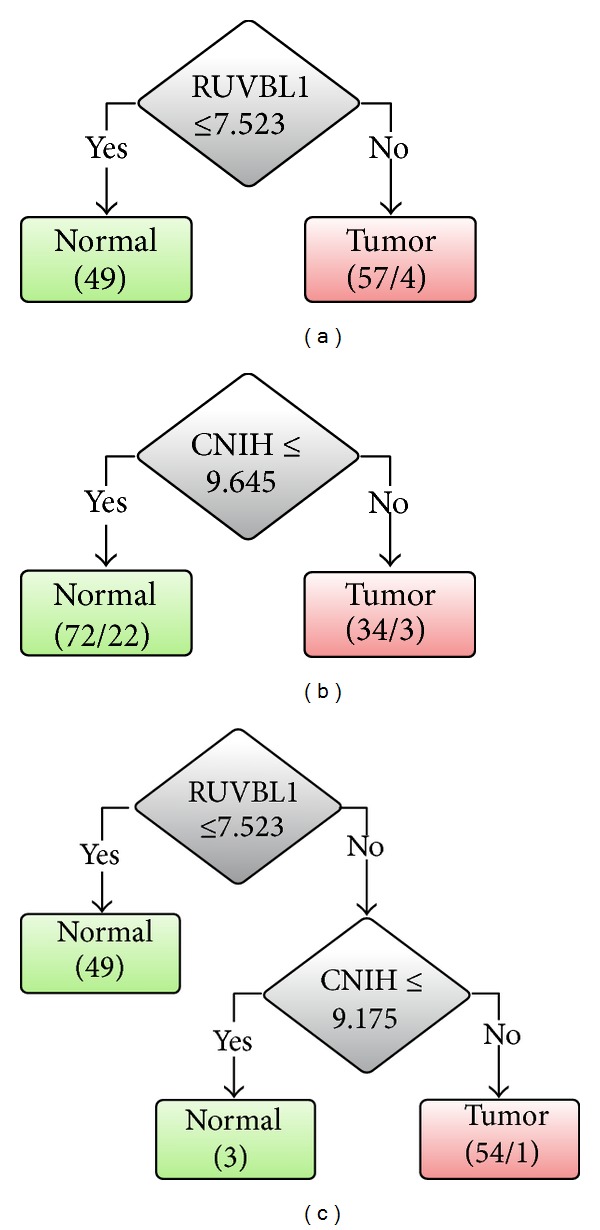
Decision tree classifiers based on GSE23400 dataset using (a) RUVBL1, (b) CNIH, and (c) both RUVBL1 and CNIH.

RUVBL1 alone can be utilized to discriminate ESCC from normal tissues. In contrast, CNIH alone is not suitable for this purpose with an LOOCV accuracy of 75.47%. However, the combined use of RUVBL1 and CNIH provides the best LOOCV accuracy. The decision tree models trained on the whole dataset GSE23400 for RUVBL1, CNIH, and both of RUVBL1 and CNIH are shown in Figure 2. The numbers shown in a tree leaf node represent the covered and misclassified samples by the corresponding decision rules. For example, in Figure 2(a), the rule of “If RUVBL1 > 7.523 then Tumor” covers 57 samples with 53 correctly classified and 4 misclassified samples. On the other hand, the rule of “If RUVBL1 ≤ 7.523 then Normal” covers 51 samples that are all correctly classified.

### 3.2. Biomarker Validation

To externally validate the two potential biomarkers of RUVBL1 and CNIH, another dataset GSE20347 fetched from GEO database consisting of 17 pairs of ESCC and normal tissues from patients in China. LOOCV is applied to GSE20347 dataset to validate the discrimination ability of RUVBL1 and CNIH for ESCC. The use of RUVBL1 yields a high accuracy of 97.06% in GSE20347 that is consistent with that in GSE23400. The same accuracy obtained from 10-CV demonstrates the usefulness of the biomarkers. However, CNIH alone failed to discriminate ESCC from adjacent normal tissues with an LOOCV accuracy of 44.18%. By using both genes of RUVBL1 and CNIH, the accuracy of the decision tree model remains unchanged.

**Figure 3:**
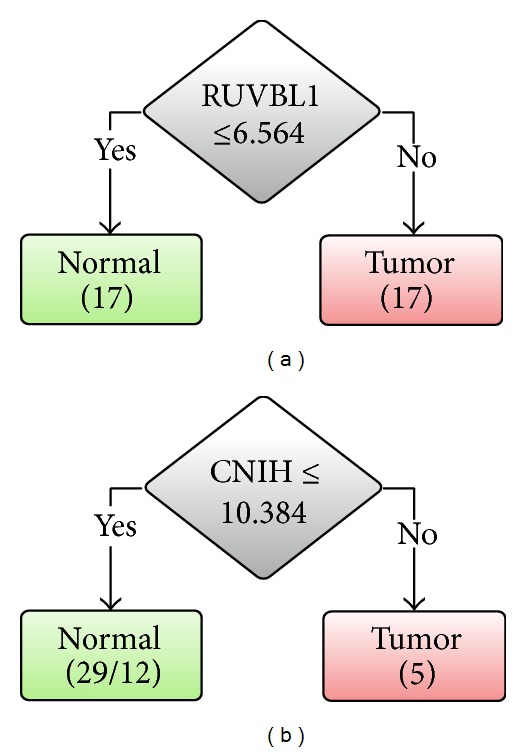
Decision tree classifiers based on GSE20347 dataset using (a) RUVBL1 and (b) CNIH.

Figures 3(a) and 3(b) show the decision tree models trained on the whole dataset of GSE20347 using RUVBL1 and CNIH, respectively. Consistent with the result of LOOCV accuracies, the decision tree models using RUVBL1 and both genes of RUVBL1 and CNIH are exactly the same. The CNIH is not utilized by the decision tree algorithm because of its poor discriminating ability.

### 3.3. Application of the Biomarkers for Predicting Esophageal Squamous Cell Carcinoma

After the identification and validation of the two biomarkers from the two public available datasets, a total of 34 gene expression profiles from 17 pairs of matched tumor and adjacent normal tissues were measured and collected to test the discriminating ability of the two biomarkers for ESCC. The 34 profiles are generated by using Human Whole Genome OneArray (HOAv4.3, Phalanx Biotech Group, Taiwan) that is different from the two datasets generated by using Affymetrix U133A chips.

The LOOCV accuracy using RUVBL1 was firstly evaluated. The sensitivity, specificity, and accuracy are 94.12%, 76.47%, and 85.29%, respectively. The performances using both genes of RUVBL1 and CNIH are 88.24%, 94.12%, and 91.18% for sensitivity, specificity, and accuracy, respectively. Results show that performances can be improved by incorporating CNIH. The LOOCV and 10-CV accuracies are exactly the same. The improvement is consistent with that in GSE23400. A comparison of classification accuracies on three datasets is shown in Table 1.

**Figure 4:**
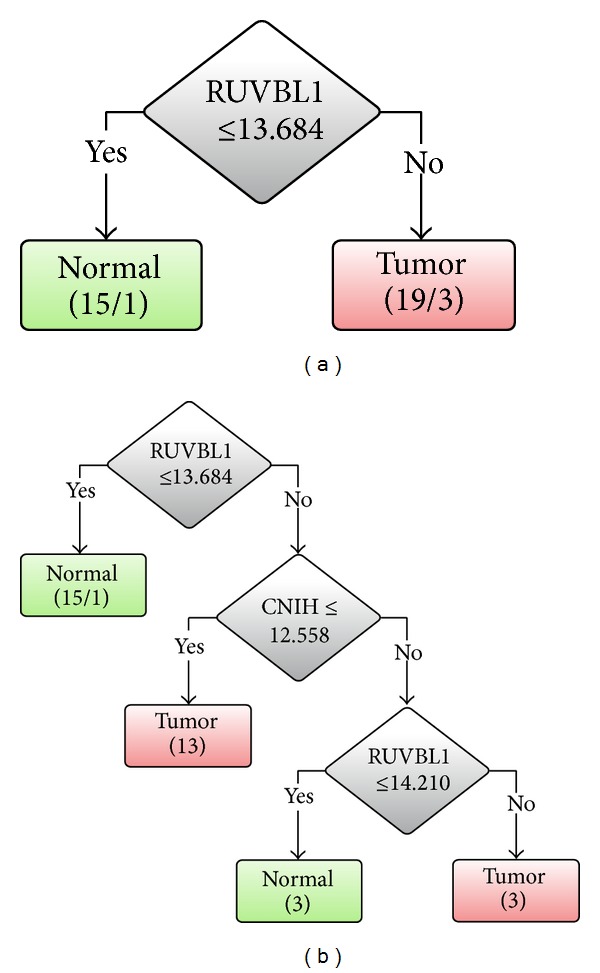
Decision tree classifiers based on our dataset using (a) RUVBL1 and (b) both RUVBL1 and CNIH.

The decision tree models trained on the 34 profiles using RUVBL1 and both genes of RUVBL1 and CNIH are shown in Figures 4(a) and 4(b), respectively. Although the same accuracy improvement is observed in both datasets of GSE23400 and 34 expression profiles of patients in China and Taiwan, respectively, it is surprising to find that CNIH is downexpressed in these Taiwan patients.

## 4. Discussion 

This study proposed a feature selection-based method to discover a small subset of genes to discriminate ESCC from normal tissues. The method was based on a sequential forward feature selection algorithm to design decision tree models for classifying expression profiles of ESCC and normal tissues. Two genes of RUVBL1 and CNIH were discovered with a high LOOCV accuracy of 99.06% in a published dataset GSE23400 (available at GEO database) consisting of 53 pairs of ESCC and normal tissues. The gene set has been validated in another dataset GSE20347 consisting of 17 pairs of ESCC and normal tissues whose platform is the same as GSE23400. A high LOOCV accuracy of 97.06% for GSE20347 shows the discrimination ability of the two genes.

To further test the two genes, microarray techniques were applied to measure gene expression profiles of Taiwanese patients. The dataset consists of 17 pairs of ESCC and normal tissues. An LOOCV accuracy of 91.18% obtained by using RUVBL1 and CNIH shows their potential as biomarkers for ESCC. Each gene alone performs worse in datasets of GSE23400 and our dataset. It suggests that the two genes should be used simultaneously to obtain the best performance. The 10-CV accuracies demonstrate that the performance remains the same when less training samples are available.

The relationship between the two newly identified biomarkers of RUVBL1 and CNIH genes and ESCC has not been reported. The decision tree models show that RUVBL1 is overexpressed in all three datasets of patients in China and Taiwan. However, CNIH is over- and downexpressed in datasets of patients in China (GSE23400) and Taiwan, respectively. For GSE20347 of patients in China, CNIH is neither over- nor downexpressed.

RUVBL1 plays important roles in chromatin remodeling, transcriptional and developmental regulation, DNA repair, and apoptosis. RUVBL1 is ubiquitously and highly expressed in thymus and testis [26–30] and interacts with major oncogenic actors such as beta-catenin and c-myc [31]. It can inhibit telomerase in cancer [32]. RUVBL1 is reported to regulate COX-2 gene expression that plays a crucial role in the progress and transformation of colon cancer [33]. Overexpression of RUVBL1 was also reported in hepatocellular carcinoma [34, 35], colorectal tumor [36], nonsmall cell lung cancer [37], B-cell lymphoma [38], and breast cancer [39]. Two SNPs in RUVBL1 were found to be associated with increased risk of serous ovarian cancer [40]. RUVBL1/2 complex can form a complex with Hsp90 and regulate phosphatidylinositol 3-kinase-related protein kinase (PIKK) family proteins [41]. PIKKs are central regulators of stress responses including DNA damage. Inhibition of Hsp90-RUVBL1/2 complex is effective for anticancer therapy [41].

CNIH is involved in the selective transport and maturation of TGF-alpha family proteins. There is no research reporting the relationship between CNIH gene and cancers. Interestingly, the expression of CNIH mRNA is affected by tetrachlorodibenzodioxin [33, 42–44]. Tetrachlorodibenzodioxin enhances the expression of CNIH mRNA in *Mus musculus* [33, 44] and inhibits CNIH expression in *Homo sapiens *[43]. Cigarette smoking is a common source of human exposure to tetrachlorodibenzodioxin and is well known as an important environmental risk factor for ESCC [45]. Also, kojic acid can increase the expression of CNIH mRNA in *Homo sapiens *[46] and is a byproduct in the fermentation process of malting rice for producing Japanese alcoholic beverage. Alcohol consumption can increase the risk of ESCC [45]. The different expressions of CNIH in ESCC tissues of the three datasets might be affected by cigarette smoking and alcohol consumption. In GSE23400, the percentages for tobacco and alcohol users were 59% and 53%, which were lower than other parts of the world. There was no data for tobacco and alcohol uses in GSE20347. In our dataset, we chose patients who were both smokers and drinkers to validate the potential biomarkers because they are the major risk factors in Taiwan and Western countries. Our previous study has shown that consumption of alcohol plus cigarettes explained 82.6% of the development of ESCC in Taiwanese males [47]. Moreover, in Linxian of China, the predominant risk factors for ESCC were diet and nutrition, instead of alcohol and smoking [48]. The diversity in major risk factors across different areas might explain the variable expression patterns of CNIH.

## 5. Conclusions

In conclusion, using feature selection and decision tree models from two public available microarray datasets, we found that two genes (RUVBL1 and CNIH), particularly RUVBL1, could be useful biomarkers in the clinic for discriminating cancer and normal tissues in Taiwanese ESCC patients. The collection of a larger dataset for independent test could further validate the robustness of the two biomarkers. A future work to study the mechanism of these two genes in the carcinogenesis of ESCC is necessary.

## Figures and Tables

**Table 1:** Classification accuracies using biomarkers of RUVBL1 and CNIH.

Biomarker	Dataset		
	GSE23400	GSE20347	17 pairs
RUVBL1	95.28%	97.06%	85.29%
RUVBL1 + CNIH	99.06%	97.06%	91.18%
